# Synthetic MR Imaging in the Diagnosis of Bacterial Meningitis

**DOI:** 10.2463/mrms.ci.2016-0082

**Published:** 2016-12-22

**Authors:** Christina Andica, Akifumi Hagiwara, Misaki Nakazawa, Kanako K Kumamaru, Masaaki Hori, Mitsuru Ikeno, Toshiaki Shimizu, Shigeki Aoki

**Affiliations:** 1 Department of Radiology, Juntendo University School of Medicine, 2-1-1 Hongo, Bunkyo-ku, Tokyo 113-8421, Japan; 2 Department of Radiology, Graduate School of Medicine, The University of Tokyo, Tokyo, Japan; 3 Department of Radiological Sciences, Graduate School of Human Health Sciences, Tokyo Metropolitan University, Tokyo, Japan; 4 Department of Pediatrics, Juntendo University School of Medicine, Tokyo, Japan

**Keywords:** synthetic MRI, magnetic resonance quantification, meningitis, meningeal enhancement

In infancy, the clinical presentation of meningitis is usually nonspecific and cerebrospinal fluid analysis is less useful.^1^ Contrast-enhanced (CE) magnetic resonance imaging (MRI) is the most sensitive imaging technique for detecting meningitis and CE T_1_ weighted imaging (T_1_WI) is the preferred sequence at many institutions.^2^ However, CE fluid attenuated inversion recovery (FLAIR) reportedly has a higher sensitivity than CE T_1_WI.^2^

Synthetic MRI is a method based on quantification of the T_1_ and T_2_ relaxation times, the proton density (PD), and the amplitude of the local radio frequency B1 field by a single scan.^3^ With this technique, tailored contrast-weighted images can be acquired with a significant reduction in examination time.

A seven-week-old female infant was hospitalized with a diagnosis of probable bacterial meningitis. Lumbar puncture was performed unsuccessfully. Blood examination showed elevated CRP and blood culture was positive for group B streptococcus.

A 3T MR system (Discovery MR750w, GE Healthcare, Milwaukee, Wisconsin, USA) with a twelve-channel head coil was used for all imaging. Synthetic images were created using SyMRI StandAlone software (SyntheticMR AB, Linköping, Sweden). The patient underwent conventional and quantitative imaging before and after intravenous administration of contrast agent (Gadoteridol 0.1 mmol/kg of body weight). CE conventional T_1_ weighted inversion recovery (T_1_IR), FLAIR, and quantitative MRI were performed 3, 8 and 16 minutes after contrast agent administration, respectively. In our institution, quantitative MRI was performed routinely in pediatric patients because of its usefulness.^4^

Parameters of synthetic non-CE and CE T_1_IR (TR 2020 ms, TE 17 ms, TI 840 ms) and FLAIR (TR 9000 ms, TE 122 ms, TI 2320 ms) were adjusted retrospectively to be the same as conventional T_1_IR (TR 2023.4 ms, TE 17.4 ms, TI 832 ms), and FLAIR (TR 9000 ms, TE 121.74 ms, TI 2472.6 ms). Non-CE conventional (Fig. 1A and B) and synthetic (Fig. 2A and B) MRI showed bilateral subdural effusion. Conventional CE T_1_IR (Fig. 1C) did not show enhancement but CE FLAIR (Fig. 1D) showed a subtle enhancement in the subdural effusion area that represents a contrast agent leakage into the effusion secondary to meningitis. Formation of the vascularized outer membrane of subdural effusion and extravasation of plasma from the blood vessels causes higher gadolinium (Gd) concentration in the effusion.^5^ Synthetic CE T_1_IR (Fig. 2C) also showed a subtle enhancement, but more obvious than conventional CE T_1_IR MR Image. More apparent enhancement on the synthetic MRI might have been caused by higher concentrations of Gd that leakage into the effusion due to longer duration time after contrast material administration. However, synthetic CE FLAIR (Fig. 2D) showed enhancement more clearly. Moreover, synthetic double IR (DIR) images also can be acquired with any combination of TI. In this case, synthetic CE DIR (TR 15000 ms. TE 100 ms, TI 260 ms, TI2 3100 ms) (Fig. 2E) showed enhancement even more clearly by nulling the CSF and minimizing the signal of fat.^3^

Bacterial meningitis is a potentially life threatening neurological emergency requiring prompt diagnosis and treatment.^2^ CE FLAIR has a high sensitivity to meningeal pathology even with low concentrations of gadolinium,^2^ but is not performed routinely. In synthetic MRI, CE FLAIR images can be easily made after the image acquisition. This case showed that synthetic CE FLAIR appears superior to conventional CE T_1_-IR and FLAIR in the diagnosis of meningitis.

## Figures and Tables

**Fig 1.**
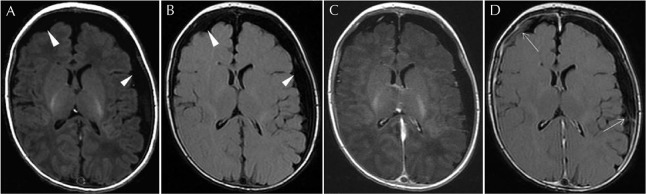
Conventional MRI. (**A**) T_1_IR and (**B**) FLAIR show subdural effusion (arrowheads). CE (**C**) T_1_IR does not show enhancement but (**D**) FLAIR shows a subtle enhancement in the subdural effusion area (arrows). MRI, magnetic resonance imaging; FLAIR, fluid attenuated inversion recovery; T_1_IR, T_1_ weighted inversion recovery.

**Fig 2.**
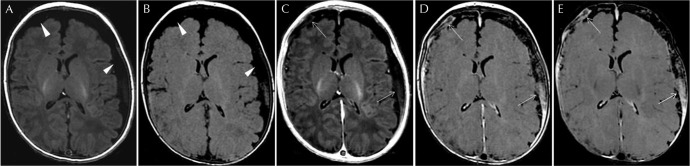
Synthetic MRI. (**A**) T_1_IR and (**B**) FLAIR show subdural effusion (arrowheads). CE (**C**) T_1_IR shows subtle enhancements (arrows). CE (**D**) FLAIR and (**E**) DIR show a clear enhancement in the subdural effusion area (arrows). MRI, magnetic resonance imaging; T_1_IR, T_1_ weighted inversion recovery; DIR, double IR; CE, Contrast-enhanced.
